# Effect of impact mechanism and intervention measures on sleep quality of college students addicted to short video: a randomly controlled trial

**DOI:** 10.3389/fnbeh.2026.1714774

**Published:** 2026-02-10

**Authors:** Jiayu Feng, Huiyan Ni, Zongping Hou, Lin Zhao, Xu Lei

**Affiliations:** 1 Department of Psychology, Southwest University, Chongqing, China; 2 Mailman School of Public Health, Columbia University in the City of New York, New York, NY, United States; 3 School of Journalism and Media, Southwest University, Chongqing, China; 4 Department of Internal Medicine, Xinxiang Central Hospital, Xinxiang, Henan Province, China

**Keywords:** algorithm recommendation, college students, intervention measures, short video addiction, sleep quality

## Abstract

**Introduction:**

The rapid growth of short video platforms has raised concerns about their impact on users’ mental health, particularly sleep quality. The aim of this research is to investigate the relationship between short video addiction and sleep quality among college students, examine the differential impacts of algorithm types (personalized vs. community-based) and content types on sleep parameters, and evaluate the effectiveness of a multi-component intervention.

**Methods:**

Sixty college students (aged 18–25) meeting criteria for short video addiction (PSQI ≥5, daily usage ≥2 h) were randomly assigned to personalized algorithm (*n* = 30) or community-based algorithm (*n* = 30) groups. Sleep quality was measured using the Pittsburgh Sleep Quality Index (PSQI), actigraphy, and the Short Video Addiction Scale (SVA-S). The intervention combined cognitive behavioral therapy (CBT), digital technology tools (time-window control, brightness adjustment), and a social support system (“Sleep Guardian”).

**Results:**

Personalized algorithms significantly worsened sleep quality compared to community-based algorithms (PSQI: 10.4 ± 2.3 vs. 8.7 ± 2.1, *p* = 0.003, Cohen’s *d* = 0.77). Entertainment content had the most detrimental effects on sleep parameters compared to knowledge and information content (*p* < 0.001, η^2^ = 0.23). The multi-component intervention significantly improved sleep quality in both groups, with PSQI scores decreasing by 3.6 points in the personalized algorithm group and 2.8 points in the community-based group (*p* < 0.001, Cohen’s *d* = 1.71 and 1.46, respectively). Daily short video usage decreased by 47.1% and 54.3%, respectively.

**Conclusion:**

Short video addiction significantly impacts sleep quality. The combination of personalized algorithms and entertainment content creates particularly detrimental conditions for sleep. A comprehensive intervention incorporating CBT, digital tools, and social support effectively improves sleep quality and reduces addiction symptoms.

## 1 Introduction

The rapid growth of short video platforms has transformed digital content consumption patterns, with significant implications for young adults’ sleep habits. According to the China Internet Network Information Center (CNNIC), by the end of 2023, China had approximately 1.0142 billion mobile internet users, with college students spending an average of 2.3 h daily on short video platforms (Jiang et al., 2024). The continuous stimulation of dopamine release through endless scrolling creates a powerful reward mechanism that encourages prolonged engagement, often extending into nighttime hours when sleep should be prioritized (Ye et al., 2025).

While previous studies have explored the relationship between digital media use and sleep disturbances, there are notable gaps in understanding the specific mechanisms through which short video addiction impacts sleep quality (Chen and Yan, 2016). Multiple systematic reviews and empirical studies have demonstrated that most research has focused broadly on “screen time” interactions without differentiating between algorithm types or content categories and their distinct effects on sleep architecture (Scott et al., 2019; Carter et al., 2016; Hale and Guan, 2015; Lian et al., 2021). For example, a meta-analysis by Carter et al. (2016) examined 20 studies on media device use and sleep outcomes in children and adolescents, finding consistent negative associations but lacking specificity regarding platform characteristics. Similarly, Hale and Guan (2015) conducted a systematic review of 67 studies and concluded that while screen-based media use was consistently associated with shorter sleep duration and poorer sleep quality, the mechanisms underlying these associations remained poorly understood. Scott et al. (2019) further emphasized that the rapid scrolling behavior and brief, high-intensity content exposure characteristic of short video platforms may create unique neurological patterns of arousal that differ significantly from traditional media consumption (Liao et al., 2024).

The relationship between short video use and sleep disruption involves multiple pathways. First, the blue light emitted by screens suppresses melatonin production, delaying sleep onset and reducing sleep quality (Ghosh et al., 2025). Second, emotionally activating content increases cognitive arousal and physiological alertness, making it difficult to transition to a relaxed state conducive to sleep (Joshi et al., 2022). Third, the displacement effect occurs when time spent on short videos directly replaces sleep time, resulting in sleep deprivation (Pirdehghan et al., 2021).

Current interventions for technology-related sleep problems have shown limited effectiveness when applied to short video consumption. Traditional approaches such as screen time limits or general digital detox strategies often fail to address the unique psychological mechanisms of short video engagement, particularly the powerful pull of personalized content algorithms (Pillion, 2023; Liao, 2024).

This research addresses these gaps by employing a technical-behavioral approach combining algorithm analysis and cognitive-behavioral therapy (CBT) principles. We investigate how different recommendation algorithms and content types affect sleep parameters, and we test the effectiveness of targeted interventions that incorporate digital technology, behavioral modification techniques, and social support systems. The study provides an integrated framework for understanding and addressing short video-related sleep disturbances among college students.

Our research has several innovative aspects. First, we examine the differential effects of personalized versus community-based recommendation algorithms on sleep quality, addressing a significant gap in understanding how algorithmic mechanisms influence sleep behavior. Second, we categorize content types (entertainment, knowledge, and information) to analyze their varying impacts on sleep. Third, we develop and test the effectiveness of a multi-component intervention that combines CBT techniques with digital tools and social reinforcement systems specifically designed for short video users.

This study aims to achieve three objectives: (1) to analyze how different algorithms (personalized and community-based) and content types affect sleep quality among college students; (2) to explore the effectiveness of a comprehensive intervention combining behavioral techniques, digital tools, and social support; and (3) to provide evidence-based recommendations for designing healthier short video platforms and developing effective interventions for improving sleep health among young adults.

## 2 Methodology

### 2.1 Participants

The study recruited 60 college students aged 18–25 years who met the practical criteria for short video addiction as measured by the Pittsburgh Sleep Quality Index (PSQI ≥5 points) and the Short Video Addiction Scale (SVA-S), with daily short video use exceeding 2 h. Participants had not previously participated in any psychological intervention related to media use or sleep problems.

The screening process involved multiple steps. First, we distributed the PSQI and SVA-S questionnaires through campus social media platforms to identify potential participants. A total of 847 students completed the initial screening questionnaires. Of these, 312 students (36.8%) met the PSQI criterion of ≥5 points, and 198 students (23.4%) also met the SVA-S criterion indicating moderate to high short video addiction. After applying the daily usage criterion (≥2 h), 127 students remained eligible. Following individual interviews to verify short video usage patterns and exclude those with pre-existing sleep disorders or psychiatric conditions, 78 students were identified as meeting all inclusion criteria. From this pool, 60 participants were randomly selected and enrolled in the study. The final sample consisted of 60 participants (37 females, 23 males) with a mean age of 20.7 years (SD = 1.6). All participants provided written informed consent, and the study was approved by the Ethics Committee of Southwest University (approval number: SWU-EC-2024-037).

To ensure sample representativeness, we intentionally recruited students from various academic disciplines, including humanities (*n* = 18), sciences (*n* = 16), engineering (*n* = 14), and arts (*n* = 12). This diversity allowed us to control for potential confounding factors related to academic workload and schedule flexibility that might influence both short video use and sleep patterns.

### 2.2 Materials

#### 2.2.1 Sleep measurement tools

*Activity monitor*: The wGT3X-BT wireless triaxial activity monitor (ActiGraph LLC, Pensacola, FL, United States) was used in combination with the ActiLife software application (version 6.13.4) to provide objective sleep measurement data (ActiGraph, 2023; Migueles et al., 2017). This device captures data on sleep status, awakening frequency, sleep time, and sleep efficiency, enabling comprehensive assessment of sleep quality. The wGT3X-BT has demonstrated excellent reliability and validity in measuring sleep–wake patterns compared to polysomnography (Slater et al., 2015).

*Pittsburgh sleep quality index* (PSQI): The PSQI is a widely used self-report questionnaire in both clinical and research settings, originally developed by Buysse et al. (1989) and validated in Chinese populations by Liu et al. (1996). It measures seven dimensions of sleep quality: subjective sleep quality, sleep latency, sleep duration, habitual sleep efficiency, sleep disturbances, use of sleep medication, and daytime dysfunction. Each dimension is scored from 0 to 3, with a total score ranging from 0 to 21. Higher scores indicate worse sleep quality, with a score of 5 or above indicating poor sleep quality. The Cronbach’s alpha coefficient for the PSQI in this study was 0.845, demonstrating good internal consistency.

*Short video addiction scale (SVA-S)*: This Chinese scale was developed by Zhang et al. (2022) at Jiangsu University in 2022 and is specifically designed to assess college students’ addiction to short videos. It measures five dimensions: compulsivity (items assessing the inability to control usage), withdrawal response (items measuring discomfort when unable to access short videos), tolerance (items indicating need for increasing usage to achieve satisfaction), consciousness (items evaluating awareness of problematic usage), and negative consequences (items assessing impact on daily life). The scale consists of 20 items rated on a 5-point Likert scale, with total scores ranging from 20 to 100. Higher scores indicate more severe addiction symptoms. The Cronbach’s alpha coefficient for the SVA-S in this study was 0.876.

#### 2.2.2 Algorithm and content models

##### 2.2.2.1 Algorithm models

*Personalized algorithm recommendation*: Using mobile applications with personalized recommendation algorithms that employ hot-start and cold-start methods to aggregate multiple signals (likes, viewing duration, etc.) to recommend short videos to users. The hot-start method refers to the algorithm’s ability to quickly adapt recommendations for users with established viewing histories by leveraging accumulated behavioral data to predict preferences (Covington et al., 2016). The cold-start method addresses the challenge of recommending content to new users or introducing new content, typically by utilizing demographic information, explicit preferences, or content-based features until sufficient behavioral data is collected (Schein et al., 2002).

*Community-based algorithm recommendation*: Using mobile applications with both “same-interest circle” and “interest connection” dual recommendation approaches, prioritizing content related to user interests and user-generated content from interconnected social networks (Yang et al., 2013).

##### 2.2.2.2 Content type models

*Entertainment*: Short video apps featuring content adjusted toward pleasure-centered videos such as comedy, dancing, and entertainment with high stimulation and low cognitive load.

*Knowledge*: Rational content featuring educational videos on knowledge, technology, and education with medium cognitive load and medium emotional stimulation.

*Information*: Rational content focusing on current affairs, social hot topics, and discussion-type videos with high information load and potentially strong emotional reactions.

#### 2.2.3 Intervention tool design

##### 2.2.3.1 Individual level

*Cognitive behavioral therapy (CBT)*: The CBT intervention was adapted from established protocols for technology addiction (Young, 2007; Winkler et al., 2013) and implemented “smartphone usage awareness training” and “sleep hygiene reinforcement” through two main approaches. First, participants were required to record their daily short video usage triggers (such as pre-bedtime boredom or emotional distress) using a reflective journal format that employed the “stop-observe-plan-act” predictive model to identify automatic behavioral patterns. Second, participants used guided imagery training in a relaxed state to reconstruct mental models about sleep (such as “imagining falling asleep in a forest while listening to birds chirping”), replacing short video usage with more sleep-conducive behaviors.

##### 2.2.3.2 Digital technology tools

*“Bring ToGo” App*: This application, developed specifically for this study based on the Digital Wellbeing framework (Google, 2023), provided time-window control, forcibly disabled app functions, and collected attention absorption data. It detected nighttime usage (23:00–6:00) and automatically adjusted screen brightness and color temperature.

*“Sleep Guardian” system*: A group-based points accumulation and incentive mechanism that connected to three consecutive sleep quality checks (via activity monitor) weekly. After achieving 50 points, participants could exchange them for rewards. Group members used short video platform message functions to motivate each other (20 points per message), with weekly rankings announcing the top 20%, creating group responsibility (such as monitoring sleep behaviors), leveraging both “offline mode” effects and social pressure to implement behavioral changes (Bandura, 1991).

### 2.3 Experimental design

The study employed a 2 × 3 × 2 factorial design. The first independent variable was algorithm type (personalized algorithm recommendation/community-based algorithm recommendation), the second was content type (entertainment/knowledge/information), and the third was intervention presence (with/without). The dependent variables were PSQI score, SVA-S score, and daily screen time.

To control for potential confounding variables, we collected data on participants’ general smartphone usage, academic workload [assessed using the Academic Stress Scale (Lin and Huang, 2014)], exercise habits, caffeine consumption, and pre-existing sleep conditions. These variables were included as covariates in the statistical analyses to isolate the effects of our experimental manipulations.

The experimental conditions were counterbalanced to minimize order effects. Participants were randomly assigned to start with either the personalized or community-based algorithm, and the content types were presented in a randomized sequence. The intervention phase followed the non-intervention phase for all participants to prevent carryover effects of the intervention strategies.

### 2.4 Procedure

**Figure 1:**
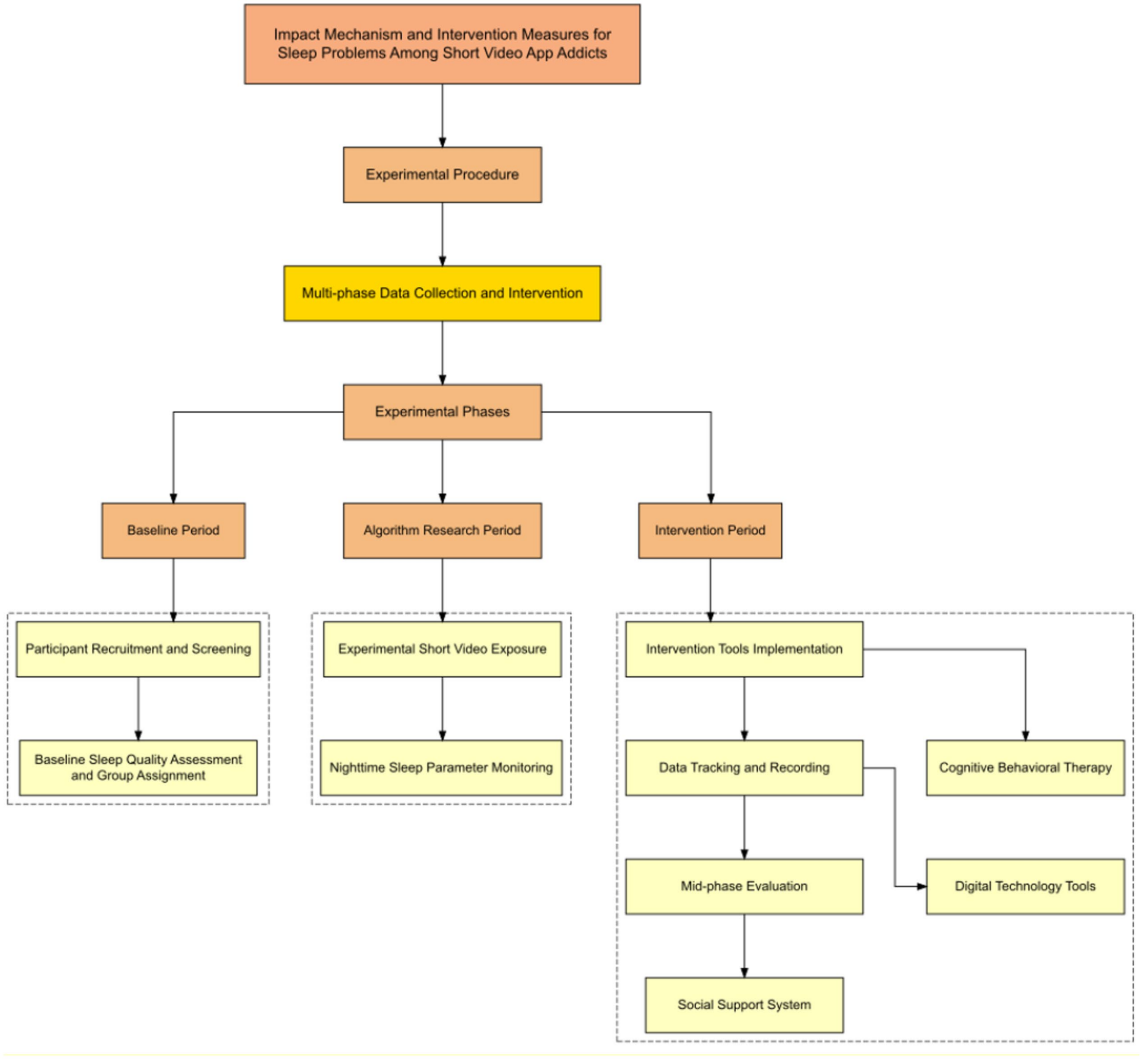
Research design flowchart for sleep problems among short video addiction.

The research spanned 8 weeks, divided into baseline, algorithm research, and intervention phases, with multiple data collection points and effectiveness verification. The detailed procedure is illustrated in Figure 1.

During the preparation phase, the research team recruited participants through university social media platforms, screened them according to the selection criteria (PSQI ≥5 points, daily short video use ≥2 h), created individual profiles for each participant, and established baseline measurements. Participants underwent continuous 7-day natural sleep monitoring using the wGT3X-BT wireless triaxial activity monitor, while simultaneously completing PSQI and SVA-S evaluations.

Next, participants were randomly assigned to either the personalized algorithm group (G1, *n* = 30) or the community-based algorithm group (G2, *n* = 30), with 10 participants assigned to each content type subgroup within each algorithm group. Both groups were provided with mobile phones pre-installed with short video applications featuring the corresponding algorithm types, but with different content type distributions. The groups used these applications for 2 weeks to establish baseline effects of algorithms and content types on sleep.

During the algorithmic research phase (weeks 2–3), baseline measurements were taken of the effects of algorithms on sleep. Participants were instructed to use the assigned short video applications for 1 h between 21:00 and 22:00 in a normally lit environment. G1 used devices with personalized algorithm recommendations, while G2 used community-based algorithms. For content distribution, 50% of G1’s content was entertainment-based “same interest circle” recommendations, while the other 50% came from “interest connection” social networks. Content type distribution followed approximately the same pattern across groups: 1/3 entertainment (comedy, dance), 1/3 knowledge (science, education), and 1/3 information (current affairs, social topics). Participants recorded sleep status (awakening frequency, sleep quality, sleep efficiency) daily, and data was regularly uploaded and analyzed.

The intervention phase (weeks 4–8) focused on validating the effectiveness of the intervention measures. All participants received CBT, including “smartphone usage awareness training” and “sleep hygiene reinforcement.” The “stop-observe-plan-act” predictive model was implemented to analyze behavior patterns, followed by four relaxation techniques for mental reconstruction of sleep concepts (such as “deep forest relaxation” and “moonlight lake meditation”). Participants also used the “Bring ToGo” app for time-window control (restricting usage between 23:00 and 6:00) and automatic brightness adjustment during evening usage times.

Additionally, the “Sleep Guardian” group incentive mechanism was established with weekly check-ins (activity monitor verification). After accumulating 50 points, rewards could be exchanged; group members motivated each other through short video platform messaging (20 points per message), with weekly rankings. In weeks 6 and 8, continuous 7-day activity monitor measurements of PSQI and SVA-S were conducted to evaluate intervention effectiveness and analyze changes in sleep participation and behavior.

Importantly, the intervention protocol included detection measures for severe sleep disturbances, with provisions to immediately stop the experiment and refer participants to professional counseling if needed, ensuring complete ethical protection for all participants.

### 2.5 Statistical analysis

All statistical analyses were performed using SPSS version 26.0 (IBM Corp., Armonk, NY, United States) and AMOS version 24.0 (IBM Corp.) for structural equation modeling. Descriptive statistics were calculated for all variables, with continuous variables expressed as mean ± standard deviation (SD) and categorical variables as frequencies and percentages.

Independent samples *t*-tests were used to compare baseline characteristics and sleep parameters between algorithm groups. One-way analysis of variance (ANOVA) with Tukey’s *post-hoc* tests was used to examine differences among content type groups. Two-way ANOVA was conducted to examine the interaction effects between algorithm types and content types on sleep parameters. Paired samples *t*-tests were used to compare pre- and post-intervention measurements within each group.

Effect sizes were calculated using Cohen’s d for pairwise comparisons (interpreted as small: 0.2, medium: 0.5, large: 0.8) and partial eta-squared (η^2^p) for ANOVA models (interpreted as small: 0.01, medium: 0.06, large: 0.14). 95% confidence intervals (CI) were reported for mean differences and effect sizes. Structural equation modeling was used to verify the intervention path model, with model fit assessed using the comparative fit index (CFI), Tucker-Lewis index (TLI), and root mean square error of approximation (RMSEA). All statistical tests were two-tailed, and the significance level was set at *p* < 0.05.

## 3 Results

### 3.1 Baseline characteristics

Table 1 presents the baseline characteristics of participants, including demographic information, initial PSQI and SVA-S scores, and short video usage patterns. No significant differences were found between algorithm groups (G1 and G2) at baseline for age, gender, PSQI scores, SVA-S scores, or daily short video usage time (all *p* > 0.05), indicating successful randomization.

**Table 1:** Baseline characteristics of participants (*N* = 60).

Characteristic	Personalized algorithm group (G1, *n* = 30)	Community-based algorithm group (G2, *n* = 30)	*p*-value
Age (years), Mean ± SD	20.8 ± 1.7	20.6 ± 1.5	0.624
Gender, *n* (%)			0.793
Male	11 (36.7%)	12 (40.0%)	
Female	19 (63.3%)	18 (60.0%)	
PSQI score, Mean ± SD	9.7 ± 2.4	9.5 ± 2.3	0.738
SVA-S score, Mean ± SD	38.4 ± 6.1	37.9 ± 5.8	0.746
Daily short video usage (hours), Mean ± SD	3.4 ± 0.9	3.5 ± 1.0	0.681
Content preferences, *n* (%)			0.916
Entertainment-focused	12 (40.0%)	11 (36.7%)	
Knowledge-focused	10 (33.3%)	11 (36.7%)	
Information-focused	8 (26.7%)	8 (26.7%)	

The average PSQI score of all participants was 9.6 ± 2.3, indicating poor sleep quality (PSQI > 5). The mean SVA-S score was 38.2 ± 5.9, suggesting moderate to high levels of short video addiction. Participants reported an average of 3.45 ± 0.95 h of daily short video use, with 38.3% preferring entertainment content, 35.0% preferring knowledge content, and 26.7% preferring information content.

### 3.2 Effects of algorithm types on sleep quality

Analysis of the algorithmic research phase revealed significant differences in sleep parameters between the personalized algorithm group (G1) and the community-based algorithm group (G2) after 2 weeks of controlled exposure, as shown in Table 2.

**Table 2:** Effects of algorithm types on sleep parameters after 2 weeks of exposure.

Sleep parameter	Personalized algorithm group (G1, *n* = 30)	Community-based algorithm group (G2, *n* = 30)	Mean difference (95% CI)	Cohen’s d (95% CI)	*p*-value
PSQI total score	10.4 ± 2.3	8.7 ± 2.1	1.7 (0.58, 2.82)	0.77 (0.24, 1.30)	0.003
Sleep latency (minutes)	42.7 ± 12.4	34.2 ± 10.8	8.5 (2.53, 14.47)	0.73 (0.20, 1.25)	0.006
Total sleep time (hours)	6.1 ± 0.7	6.7 ± 0.6	−0.6 (−0.94, −0.26)	0.92 (0.38, 1.46)	0.001
Sleep efficiency (%)	78.3 ± 5.4	84.1 ± 4.9	−5.8 (−8.45, −3.15)	1.12 (0.57, 1.68)	<0.001
Awakenings during night	4.2 ± 1.3	3.1 ± 1.0	1.1 (0.51, 1.69)	0.95 (0.41, 1.49)	0.001
SVA-S score	41.2 ± 5.4	36.4 ± 5.1	4.8 (2.10, 7.50)	0.91 (0.37, 1.45)	0.001
Pre-sleep arousal (1–10)	6.8 ± 1.2	5.3 ± 1.1	1.5 (0.91, 2.09)	1.30 (0.74, 1.87)	<0.001

*p < 0.05, statistically significant difference; CI, confidence interval.

Participants in the personalized algorithm group (G1) showed significantly worse sleep quality across all measured parameters compared to those in the community-based algorithm group (G2). The PSQI scores increased from baseline in G1 but decreased slightly in G2. Sleep latency was approximately 25% longer in G1, while total sleep time was shorter by an average of 36 min. Sleep efficiency was notably lower in G1 (78.3% vs. 84.1%), and participants in G1 experienced more nighttime awakenings.

The SVA-S scores increased in G1 but decreased slightly in G2, suggesting that personalized algorithms intensified addiction symptoms while community-based algorithms had a more neutral or slightly positive effect. Pre-sleep arousal levels, measured on a scale of 1–10, were significantly higher in G1, indicating greater difficulty transitioning to sleep after exposure to personalized algorithm content.

### 3.3 Effects of content types on sleep quality

Table 3 presents the effects of different content types on sleep parameters, regardless of algorithm group assignment.

**Table 3:** Effects of content types on sleep parameters.

Sleep parameter	Entertainment content (*n* = 20)	Knowledge content (*n* = 20)	Information content (*n* = 20)	*F*-value	η^2^p (95% CI)	*p*-value
PSQI total score	11.2 ± 2.4^a^	8.9 ± 2.0^b^	9.5 ± 2.2^b^	6.92	0.20 (0.04, 0.35)	0.002
Sleep latency (minutes)	45.3 ± 13.1^a^	33.7 ± 9.8^b^	36.6 ± 10.5^b^	7.38	0.21 (0.05, 0.36)	0.001
Total sleep time (hours)	5.9 ± 0.8^a^	6.6 ± 0.5^b^	6.5 ± 0.6^b^	7.13	0.20 (0.04, 0.35)	0.002
Sleep efficiency (%)	76.2 ± 5.8^a^	85.7 ± 4.5^b^	82.0 ± 5.1^c^	16.42	0.37 (0.17, 0.50)	<0.001
Awakenings during night	4.7 ± 1.4^a^	2.8 ± 0.9^b^	3.5 ± 1.1^c^	15.69	0.36 (0.16, 0.49)	<0.001
SVA-S score	43.5 ± 5.6^a^	34.7 ± 4.8^b^	38.3 ± 5.2^c^	14.92	0.34 (0.14, 0.48)	<0.001
Pre-sleep arousal (1–10)	7.4 ± 1.3^a^	4.9 ± 1.0^b^	6.0 ± 1.2^c^	25.73	0.47 (0.27, 0.59)	<0.001

*p < 0.05, statistically significant difference; ^a,b,c^Different superscripts indicate significant differences between groups (*p* < 0.05) in *post-hoc* tests; η^2^p, partial eta-squared; CI, confidence interval.

One-way ANOVA revealed significant differences in all sleep parameters across the three content types, with large effect sizes (η^2^p ranging from 0.20 to 0.47). Post-hoc comparisons using Tukey’s HSD test showed that entertainment content had the most detrimental effects on sleep quality. Participants exposed to entertainment content had significantly higher PSQI scores, longer sleep latency, shorter total sleep time, lower sleep efficiency, more nighttime awakenings, higher SVA-S scores, and higher pre-sleep arousal compared to those exposed to knowledge or information content.

Knowledge content was associated with the best sleep outcomes, with participants showing the lowest PSQI scores, shortest sleep latency, longest total sleep time, highest sleep efficiency, fewest nighttime awakenings, lowest SVA-S scores, and lowest pre-sleep arousal. Information content produced intermediate effects, significantly better than entertainment content but worse than knowledge content for most parameters.

### 3.4 Interaction between algorithm types and content types

A two-way ANOVA revealed significant interaction effects between algorithm types and content types on sleep parameters, as shown in Table 4.

**Table 4:** Interaction effects between algorithm types and content types on PSQI scores.

Algorithm type	Entertainment content	Knowledge content	Information content
Personalized (G1)	12.6 ± 2.5	9.4 ± 2.1	10.2 ± 2.3
Community-Based (G2)	9.8 ± 2.2	8.4 ± 1.9	8.8 ± 2.0

Interaction *F*-value = 3.87, *p* = 0.026*, η^2^p = 0.13 (95% CI: 0.01, 0.27).

*p < 0.05, statistically significant interaction effect.

The interaction effect indicates that the impact of content type on sleep quality was moderated by the algorithm type. The negative effects of entertainment content were amplified when delivered through personalized algorithms, resulting in substantially worse sleep quality (PSQI = 12.6) compared to when delivered through community-based algorithms (PSQI = 9.8). This pattern was consistent across all sleep parameters, suggesting that the combination of personalized algorithms and entertainment content creates a particularly detrimental condition for sleep health.

### 3.5 Effectiveness of intervention measures

Table 5 presents the changes in sleep parameters and short video addiction scores from baseline to post-intervention for both algorithm groups.

**Table 5:** Changes in sleep parameters and SVA-S scores after intervention.

Parameter	Personalized algorithm group (G1, *n* = 30)			Community-based algorithm group (G2, *n* = 30)		
	Pre-intervention	Post-intervention	Cohen’s d (95% CI)	Pre-intervention	Post-intervention	Cohen’s d (95% CI)
PSQI total score	10.4 ± 2.3	6.8 ± 1.9	1.71 (1.10, 2.31)	8.7 ± 2.1	5.9 ± 1.7	1.46 (0.88, 2.05)
Sleep latency (minutes)	42.7 ± 12.4	28.3 ± 8.7	1.34 (0.77, 1.92)	34.2 ± 10.8	25.1 ± 7.9	0.96 (0.42, 1.50)
Total sleep time (hours)	6.1 ± 0.7	7.0 ± 0.5	1.48 (0.89, 2.06)	6.7 ± 0.6	7.3 ± 0.4	1.18 (0.62, 1.73)
Sleep efficiency (%)	78.3 ± 5.4	86.5 ± 4.2	1.70 (1.09, 2.30)	84.1 ± 4.9	89.2 ± 3.8	1.16 (0.60, 1.71)
Awakenings during night	4.2 ± 1.3	2.6 ± 1.0	1.38 (0.80, 1.96)	3.1 ± 1.0	2.2 ± 0.8	0.99 (0.45, 1.54)
SVA-S score	41.2 ± 5.4	32.7 ± 4.8	1.66 (1.06, 2.26)	36.4 ± 5.1	30.2 ± 4.5	1.29 (0.72, 1.86)
Daily short video usage (hours)	3.4 ± 0.9	1.8 ± 0.7	1.98 (1.34, 2.62)	3.5 ± 1.0	1.6 ± 0.6	2.31 (1.62, 2.99)

*p < 0.001, statistically significant difference; CI, confidence interval.

Paired *t*-tests revealed significant improvements in all sleep parameters and addiction scores for both algorithm groups after the four-week intervention period, with uniformly large effect sizes (Cohen’s d ranging from 0.96 to 2.31). The PSQI scores decreased by 3.6 points (95% CI: 2.9, 4.3) in G1 and 2.8 points (95% CI: 2.2, 3.4) in G2, bringing both groups closer to the cutoff for good sleep quality (PSQI < 5). Sleep latency decreased by approximately 14 min in G1 and 9 min in G2. Total sleep time increased by about 54 min in G1 and 36 min in G2.

Sleep efficiency improved to 86.5% in G1 and 89.2% in G2, surpassing the generally accepted threshold for good sleep efficiency (85%). The number of nighttime awakenings decreased by 1.6 in G1 and 0.9 in G2. SVA-S scores decreased by 8.5 points in G1 and 6.2 points in G2, indicating substantial reductions in short video addiction symptoms.

The most dramatic change was in daily short video usage time, which decreased by 47.1% in G1 (from 3.4 to 1.8 h) and 54.3% in G2 (from 3.5 to 1.6 h). This reduction suggests that the intervention was highly effective in modifying short video consumption behavior.

### 3.6 Differential effectiveness of intervention components

To understand the relative contribution of different intervention components, participants rated the perceived effectiveness of each component on a scale of 1–10. Table 6 presents these ratings along with objective measures of adherence to each component.

**Table 6:** Perceived effectiveness and adherence to intervention components.

Intervention component	Effectiveness rating (1–10)	Adherence rate (%)
CBT—Usage awareness training	7.8 ± 1.5	82.5
CBT—Sleep hygiene reinforcement	8.3 ± 1.3	78.9
Digital tool—Time window control	9.1 ± 0.9	94.3
Digital tool—Brightness adjustment	6.7 ± 1.7	96.8
“Sleep Guardian” system—Points accumulation	8.5 ± 1.2	88.7
“Sleep Guardian” system—Group messaging	7.9 ± 1.6	73.2

The time window control feature of the digital tool received the highest effectiveness rating (9.1/10) and had a high adherence rate (94.3%), suggesting that restricting short video use during specific time windows (especially 23:00–6:00) was particularly effective for improving sleep. The automatic brightness adjustment feature had the highest adherence rate (96.8%) but a relatively lower effectiveness rating (6.7/10), indicating that while this feature was consistently used, participants perceived it as less impactful for improving sleep.

The “Sleep Guardian” points accumulation system was rated highly effective (8.5/10) with good adherence (88.7%), while the group messaging component had lower adherence (73.2%) but was still perceived as moderately effective (7.9/10). Within the CBT components, sleep hygiene reinforcement was rated more effective than usage awareness training (8.3 vs. 7.8), although adherence was slightly lower.

### 3.7 Path analysis of intervention effectiveness

**Figure 2:**
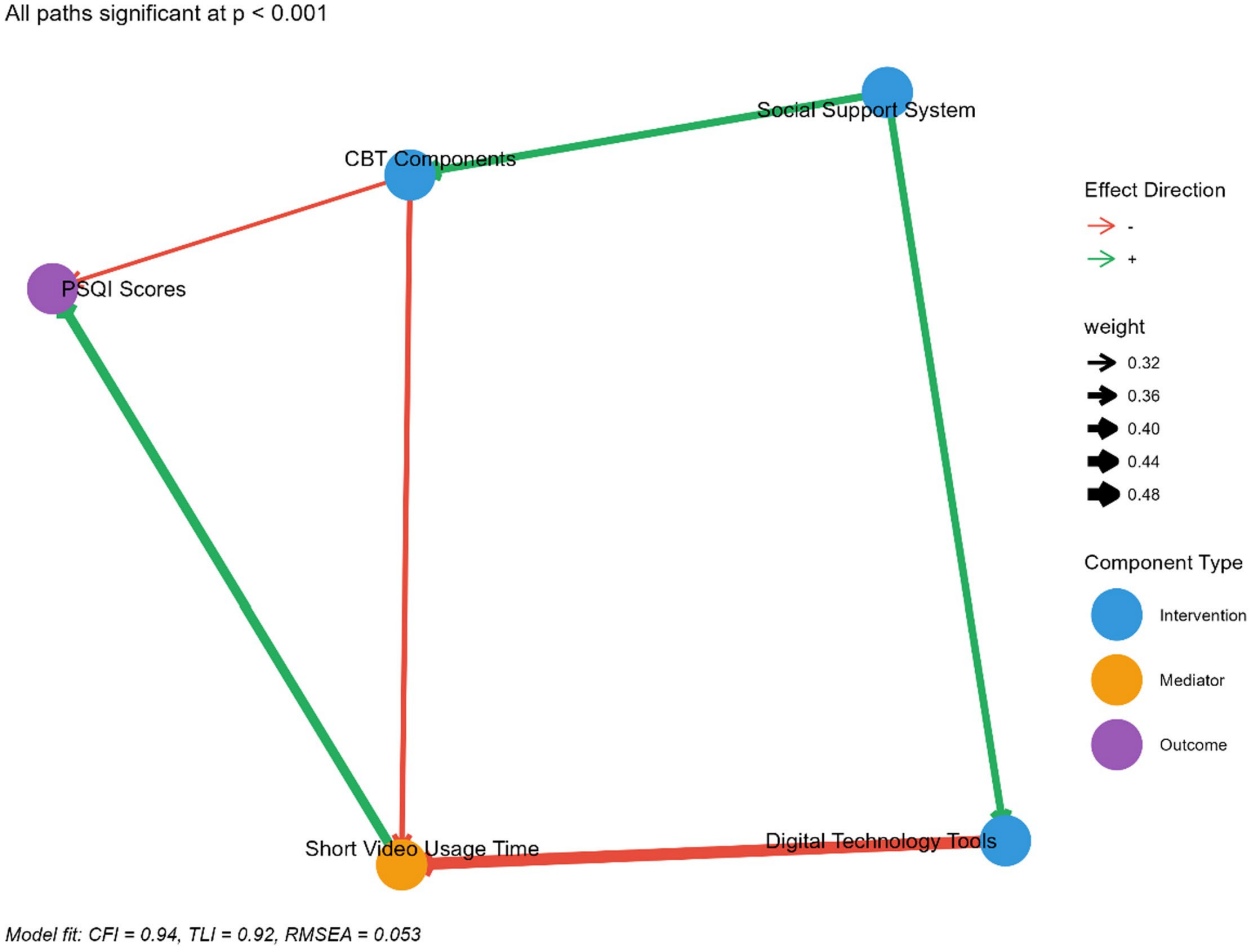
Standardized path coefficients for the intervention model.

Structural equation modeling was used to analyze the pathways through which the intervention components influenced sleep quality. The model demonstrated good fit (CFI = 0.94, TLI = 0.92, RMSEA = 0.053, 90% CI: 0.032, 0.074), and the standardized path coefficients are presented in Figure 2.

The model revealed that digital technology tools had the strongest direct effect on reducing short video usage time (*β* = −0.48, *p* < 0.001, 95% CI: −0.62, −0.34), which in turn had a significant effect on improving PSQI scores (*β* = 0.43, *p* < 0.001, 95% CI: 0.28, 0.58). CBT components had both direct effects on PSQI scores (*β* = −0.32, *p* < 0.001, 95% CI: −0.47, −0.17) and indirect effects through reduced short video usage (*β* = −0.35, *p* < 0.001, 95% CI: −0.50, −0.20). The social support system primarily worked through enhancing adherence to the other components (*β* = 0.39, *p* < 0.001, 95% CI: 0.24, 0.54) rather than directly affecting sleep outcomes.

The path analysis also indicated that the effectiveness of the intervention differed based on the algorithm type participants were exposed to during the experimental phase. The intervention had a stronger effect on reducing PSQI scores for participants in the personalized algorithm group (total effect = −3.6, 95% CI: −4.3, −2.9) compared to the community-based algorithm group (total effect = −2.8, 95% CI: −3.4, −2.2), suggesting that those exposed to more addictive algorithmic patterns benefited more from the structured intervention.

### 3.8 Mediating mechanisms

To further explore the mechanisms through which the intervention improved sleep quality, we conducted mediation analyses examining whether reductions in pre-sleep arousal and short video usage time mediated the intervention effects on PSQI scores. The analysis revealed that reduced short video usage time significantly mediated the relationship between the intervention and improved PSQI scores (indirect effect = −1.42, 95% CI: −1.89, −0.95, *p* < 0.001), accounting for 44.3% of the total effect. Additionally, reduced pre-sleep arousal served as a significant mediator (indirect effect = −0.87, 95% CI: −1.28, −0.46, *p* < 0.001), accounting for 27.2% of the total effect. Together, these two mediators explained 71.5% of the intervention’s effect on sleep quality improvement, while the remaining 28.5% was attributable to the direct effect of the intervention components on sleep processes.

## 4 Discussion

This study investigated the impact mechanisms of short video addiction on sleep problems among college students and tested the effectiveness of a multi-component intervention designed to address these problems. Our findings provide several important insights for understanding and addressing the sleep-related consequences of short video use.

First, our results demonstrate that algorithm type significantly influences sleep quality, with personalized algorithms having a more detrimental effect than community-based algorithms. The effect sizes for these differences were medium to large (Cohen’s d ranging from 0.73 to 1.30), indicating clinically meaningful distinctions between algorithm types. This finding is consistent with previous research suggesting that personalized recommendation systems are designed to maximize engagement by leveraging individual preferences and usage patterns, potentially creating stronger addiction patterns (Thomée et al., 2011; Hysing et al., 2015; Chaudhary et al., 2022; Karaman and Arslan, 2024; Atalatti and Pawar, 2024; Montag et al., 2019; Alter, 2017). The continuous feed of highly targeted content based on previous viewing behavior likely promotes the “just one more video” phenomenon that extends usage into sleep hours.

Second, our analysis of content types revealed that entertainment content had the most negative impact on sleep parameters, followed by information content, while knowledge content had the least detrimental effects. The large effect sizes observed (η^2^p ranging from 0.20 to 0.47) underscore the substantial influence of content type on sleep outcomes. This pattern may be explained by the different levels of cognitive and emotional arousal associated with each content type. Entertainment videos often feature high-stimulation content designed to elicit emotional responses, potentially increasing physiological arousal and making it more difficult to transition to sleep (Lemola et al., 2015; Christensen et al., 2016; Setyowati et al., 2023; Zhang et al., 2024; Cain and Gradisar, 2010). Knowledge-focused content, while engaging, may involve more structured cognitive processing that is less likely to create the emotional excitement that disrupts sleep architecture.

The significant interaction between algorithm type and content type (η^2^p = 0.13) is particularly noteworthy. The combination of personalized algorithms with entertainment content created a substantially worse condition for sleep compared to other combinations. This synergistic negative effect suggests that personalized delivery of highly stimulating content may create a particularly powerful form of engagement that is especially disruptive to sleep processes. This finding extends previous work by Liu et al. (2019), who examined digital media effects on sleep but did not specifically address the interaction between delivery mechanisms and content types.

Our multi-component intervention demonstrated significant effectiveness in improving sleep quality and reducing short video addiction symptoms across both algorithm groups, with uniformly large effect sizes (Cohen’s d ranging from 0.96 to 2.31). The comprehensive approach combining CBT techniques, digital tools, and social support appears to address multiple aspects of the problem simultaneously. The CBT components likely helped participants recognize and modify problematic usage patterns, while the digital tools provided structural support by creating environmental constraints on usage. The social support system enhanced motivation and accountability, addressing the social reinforcement aspects of short video use.

The mediating mechanism analysis revealed that the intervention worked primarily by reducing short video usage time (accounting for 44.3% of the total effect) and decreasing pre-sleep arousal (accounting for 27.2% of the total effect). These findings suggest that effective interventions should target both behavioral modification (reducing usage) and physiological regulation (reducing arousal) to maximize sleep improvements. The remaining direct effect (28.5%) may reflect the influence of improved sleep hygiene practices and cognitive restructuring that directly enhance sleep quality independent of usage reduction.

The differential effectiveness of intervention components provides insights for designing targeted interventions. The strong effectiveness ratings for time window control suggest that creating technological boundaries around usage times may be particularly important for addressing sleep-related problems. This aligns with Owens et al. (2014) findings that technological restrictions during pre-sleep hours can significantly improve sleep onset and quality. The high adherence to brightness adjustment, despite lower perceived effectiveness, indicates that passive interventions requiring minimal effort from users may achieve better compliance, even if their direct impact is more modest.

The finding that intervention effectiveness was greater for participants previously exposed to personalized algorithms has important implications. It suggests that individuals using platforms with more addictive algorithmic designs may benefit more from structured interventions, possibly because they have developed stronger usage habits that require more comprehensive approaches to modify. This differential response pattern should inform the targeting and design of future interventions, potentially prioritizing users of platforms known to employ highly engaging algorithmic systems (Fuller et al., 2017).

Several limitations of this study should be acknowledged, and these should be considered when interpreting our findings. First, the eight-week duration, while sufficient to demonstrate immediate effects, does not provide information about long-term sustainability of improvements. Given that behavioral changes often attenuate over time, future research should include longer follow-up periods (e.g., 6–12 months) to assess whether the positive changes persist after intervention cessation and whether booster sessions are needed to maintain gains. Second, although we controlled for several potential confounding variables including academic workload, exercise habits [consistent with findings that physical activity influences mobile phone addiction and sleep quality (Wang et al., 2025; Wang et al., 2024; Wang et al., 2024; Yang et al., 2021; Yang et al., 2022)], and caffeine consumption, other factors such as psychological stress, underlying anxiety or depression, relationship difficulties, and social dynamics may have influenced both short video use and sleep patterns. Future studies should employ more comprehensive baseline assessments of mental health status and consider these factors in multilevel analyses. Third, our sample consisted exclusively of college students from a single university in China, limiting generalizability to other age groups, educational backgrounds, cultural contexts, or populations with different usage patterns. The homogeneity of our sample, while beneficial for internal validity, restricts the external validity of our findings.

Fourth, self-selection bias may have influenced our results, as participants who volunteered for a study on short video addiction and sleep may have been more motivated to change their behaviors than the general population of short video users. This heightened motivation could have inflated our intervention effect sizes. Fifth, the potential for Hawthorne effects should be acknowledged, as participants’ awareness of being observed may have influenced their behavior independent of the intervention components. The use of objective actigraphy data partially mitigates this concern, but subjective measures such as PSQI scores may still be affected by social desirability bias. Sixth, our study design did not include a no-intervention control group, which limits our ability to attribute all observed improvements specifically to the intervention rather than to natural fluctuations, regression to the mean, or placebo effects.

Despite these limitations, our study makes several significant contributions to understanding and addressing short video-related sleep problems. By differentiating between algorithm types and content categories, we provide a more nuanced understanding of how specific aspects of short video platforms affect sleep. Our multi-component intervention demonstrates that a comprehensive approach targeting both usage behaviors and underlying psychological factors can effectively improve sleep quality among short video addicts.

These findings have important implications for multiple stakeholders. For platform designers and policymakers, the results highlight the potential sleep-related consequences of certain algorithmic designs and content promotion strategies. Our findings suggest the need for platform-level interventions such as mandatory usage limits during night hours, reduced promotion of highly stimulating content in evening time slots, and the implementation of “digital wellbeing” features that nudge users toward healthier consumption patterns. Regulatory frameworks might consider requiring platforms to disclose the potential health impacts of their algorithmic systems and to provide users with greater control over recommendation settings. Additionally, platforms could be encouraged to develop and promote sleep-friendly modes that automatically activate during nighttime hours, limiting access to high-stimulation content and reducing the reinforcing properties of personalized recommendations.

For health professionals, our intervention model provides a template for addressing short video-related sleep problems through a combination of behavioral, technological, and social approaches. The strong effect sizes observed suggest that structured interventions can meaningfully improve sleep outcomes even among individuals with established problematic usage patterns. Clinical practitioners should consider screening for short video addiction in patients presenting with sleep complaints and should be prepared to incorporate technology-specific strategies into standard CBT-I protocols.

For users themselves, understanding the differential impacts of algorithm types and content categories can inform more conscious choices about platform use and content consumption, particularly during pre-sleep hours. Our findings suggest that users may benefit from actively selecting community-based recommendation settings where available, limiting entertainment content consumption in the hours before sleep, and utilizing built-in screen time management features.

Future research should explore the long-term effects of different algorithm types on sleep and examine whether personalized algorithms create more persistent sleep disturbances compared to community-based approaches. Additionally, investigations of potential age and developmental differences in susceptibility to algorithm-induced sleep problems would provide valuable insights for designing age-appropriate interventions. Younger adolescents may be particularly vulnerable to these effects given ongoing brain development and less established self-regulatory capacities. Furthermore, collaborative research with platform developers could explore the feasibility and effectiveness of integrating sleep-protective features directly into short video applications, potentially creating platforms that balance engagement with user wellbeing.

In conclusion, this study demonstrates that short video addiction significantly impacts sleep quality among college students, with effects moderated by algorithm type and content categories. Our findings highlight the particular concerns associated with personalized algorithms and entertainment content, while also providing evidence for the effectiveness of a multi-component intervention approach. As short video platforms continue to grow in popularity, understanding and addressing their impacts on sleep becomes increasingly important for protecting the health and wellbeing of young adults.

## Data Availability

The raw data supporting the conclusions of this article will be made available by the authors, without undue reservation.
